# Effectiveness of embodied evaluation of mobile applications: A qualitative study

**DOI:** 10.1016/j.heliyon.2023.e17043

**Published:** 2023-06-08

**Authors:** Júlia Carneiro de Almeida Nogueira, Alex Sandro Gomes, Amadeu Sá de Campos Filho, Fernando Moreira

**Affiliations:** a Centro de Informática - CIn - UFPE, Brazil; b REMIT, IJP, Universidade Portucalense & IEETA, Universidade de Aveiro, Portugal

**Keywords:** Mobile computing, Prototyping, Embodied assessment technique, Usability evaluation, Human-computer interaction, User-centered design

## Abstract

As a consequence of technological advances, the number of devices has increased, with the emergence of different smartphones, tablets, and smartwatches present in both personal and professional activities. As a result, mobile applications have been developed, and with them comes the need for techniques and methods that provide support for conducting evaluation and prototyping since the current approaches are limited and cannot support the complexity and the need for understanding the context. The overall goal of the study is to evaluate the applicability of adopting a situated and embodied approach to mobile application usability testing. The aspects of postmodern and phenomenological approaches were taken into consideration. The study was conducted using the technique of digital ethnography, in particular the re-enactment technique, combined with qualitative research techniques to capture the evidence that the situated and embodied approach allows for capturing the perceptions and experience of the participants about the use of the application under evaluation. As motivation, there is the theoretical and methodological evolution and contribution and the proof that the postmodern and situated approach allows us to evaluate the usability of mobile applications in a complete way, considering the context and the user experience. The results show aspects of experience, reflections, perceptions, contingencies, practices, and meanings that go beyond the complexity of interactions and context with the use of the application, reinforcing the effectiveness of the use of the situated prototyping approach.

## 1 Introduction

Product designs are not always easy to develop. An inadequate design can make an individual less attracted, frustrated, or even unable to complete an activity [1]. User experience is fundamental to the process of product creation and success. Therefore, Human-Computer Interaction (HCI) is a dynamic and interdisciplinary area concerned with the design, evaluation, and creation of new products. Through it, it is possible to understand the relationship between human use, technologies, and phenomena [2]. User-Centered Design approaches are fundamental in identifying the main needs and problems of a new product with its main stakeholders.

As the main ally of these approaches and a source of resources for evaluating and refining a system, we have the use of prototypes. According to Ref. [3], prototyping is a strategy used by the human-computer interaction area to solve problems associated with traditional software development approaches. Given the evolutionary view of software development, prototyping can cause impacts throughout the process since it is possible to adopt the approach of software construction supported by experiments and experiences [3]. Through prototyping, it is possible to experience an initial developed version in less time and with less financial resources [4].

Although there are many approaches to usability, application evaluation, and design patterns, the use of prototyping tools and methodologies for mobile devices and applications is still an incipient area due to the lack of consensus on methodological approaches [5].

According to Ref. [6], the evaluation of applications has a number of restrictions and challenges, such as the lack of guidelines, absence of metrics and defined criteria, technological support that can capture and collect relevant data on usability, and approaches that consider the use of the application in real-time. The reason for this is due to the usage of the characteristics since a mobile device/application can be used in different environments, scales and screen sizes, by factors of the multiplicity of activities during use, and by the intrinsic limitations of users and usability [[7], [8], [9], [10], [11][[12], [13]]]. As a result, no author has introduced a formal definition of usability for mobile devices and applications. Current studies define usability through existing general norms and standards, such as ISO 9241–11 [5].

In the field of literature, there's an increasing embrace of anthropological methods to study human-computer interaction. This trend mirrors the third wave of HCI, which underscores the importance of observing the context and the ability to assess the relationships between elements. It's characterized by the diverse nature of the interaction, stemming from the growth in types of devices, the context's usage beyond just the workplace, as well as emotions and experiences that are unique, subjective, or virtual, influenced by social, political, cultural, economic, ethnic, and gender values. These aspects have not been thoroughly explored in previous research [[14], [15], [16], [17], [18]].

From this perspective, it is possible to understand the emergences of everyday activities, their unpredictability, and the meanings underlying the indeterminate actions of improvisations and the sensory forms observed between users' experience and interaction with technology [19].

According to Ref. [20], during user interaction with a product in a specific context, social and cultural factors influence the user experience [1]. Exposes that interactions, however simple, it is possible to observe complex relationships that go beyond and are interdependent of humans and non-animate objects. Against that background, in situ or situated approaches are promising for studying subjective and dynamic phenomena resulting from the experience and interaction with an application in the context of use [21]. The reason for this is the need to consider contextual interactions, dynamic environment changes and define the relevant aspects for interaction using a static evaluation approach [22]. Facing concepts of ubiquitous computing and active systems, interaction with computer systems goes beyond the use of a physical device such as a keyboard or touch screen. As new technologies emerge, the concepts of embodiment become more relevant [23].

Classical evaluations are approaches that do not consider users' senses of device use. The focus of the analyses is on objective aspects, observable and measurable aspects. Usability evaluations conclude on the ease or otherwise of use of the application without recognizing the senses that the artifacts have for users in the context of the activities.

Anthropological approaches to design have borrowed from HCI the ethnographic and phenomenological methods of analysis and evaluation of interactions and evolution of technologies [24,25]. In view of this perspective, it is possible to understand the emergences of everyday activities, their unpredictability, and the meanings underlying the indeterminate actions of improvisations and the sensory forms observed between users' experience and interaction with technology [19]. In this way, contextual, holistic, and subjective involvement are reinforced with contextual awareness of the environment, and thus actions are readily performed in a natural way [1].

The primary objective of this study is to assess the feasibility of the situated and embodied approach in the usability testing of mobile applications. In this context, a mobile application known as MobCare was utilized, designed to track children with Microcephaly and consolidate information pertaining to clinical conditions. By doing so, it will enable us to observe how the adoption of a postmodern and phenomenological approach, facilitated by Digital Ethnography, allows for the transcendence of aspects typically observed through a Cartesian approach in usability evaluation.

The organization of this article is as follows: an initial presentation of a concise theoretical framework. This is followed by an in-depth description of the methodology, the research problem, and the choices of participants. Subsequently, a brief overview of the data collection and analysis process is given. The article culminates with a discussion of the research's findings and conclusions.

## 2 Situated and embodied prototyping in mobile applications

Evaluation is a crucial component of the design procedure. Its main goal is to improve the design of a system, both in the usability aspects and in the users' experiences when interacting with them. Its activities consist of collecting and analyzing data about users' or likely users' experiences with a draft screen, prototype, application, or computer system [26].

From the mobile HCI point of view, the discussion is mainly centered on “where” and “how” to do the evaluations, with the differentiation between field and laboratory studies. Field studies comprise studies conducted in the real world, with characteristics of the social and cultural context. These approaches commonly collect data through observations, interviews, and questioning/surveys. They allow obtaining rich data with a high level of ecological validity but may be subject to bias and a low level of control that can impact validity and generalizability. In laboratory studies, the collection instruments include video recording, registers, and questionnaires, and the phenomena are analyzed in an artificial and controlled environment. Its advantages include the ability to focus on details, high replicability, and experimental control, but it has a low level of ecological validity [27].

Situated design approaches arise from postmodern paradigms of human development. In this paradigm, the participation of users in the design process becomes active, and they are no longer observed. The design seeks to live with them in real contexts of use by recognizing their identity, try to apprehend the meaning of their practices, and thus identify the first insights for the design process. Through the study of human action, it is possible to recover mental constructs such as beliefs, desires, intentions, symbols, ideas, schemes, planning, and problem-solving. It is possible because Cognitive Science approaches contribute to the activities of designers by describing the interaction between people and machines. It occurs due to the evolution of technologies in which it is no longer a mechanical interaction, such as pushing a button, for the observation of behaviors and the socialization of artifacts [28].

As a result of advances in cognitive science, Embodied Cognition emerges as a new paradigm of situated cognition [29].

Given this, Embodied Cognition points out that physical activities and lived experience gained through movement, visual connections, and the physical body provide learning that is greater than just thinking [29,30].

[31] Argues that human cognition is formed by sensory-motor processing. The cognition is governed by the actual events and pressure of interaction, which can be influenced by the settings of the environment. Furthermore, interaction is a result of cognitive aspects obtained and stored previously by body memory. Therefore, as an individual move in an environment, new perceptions can be obtained, which can replace old observations [32] such that it can be stated that humans learn about the world and its properties by interacting within it [33].

Situated approaches and the notion of embodiment bring to HCI a new trajectory [34]. It allows analyzing through prototyping the ideas and specifications of the developed design [24], as well as identifying how people feel and think through bodily feedback [35,36]. The embodiment can also help in understanding the material and non-material relationships that impact the experience resulting from the interaction between a person and a technological artifact [37[[38], [39]]]. It occurs due to the awareness of context, as it allows the export in different conditions and states. As a result, it is possible to understand and discover ways of application, values, and relationships through experienced situations [40].

In sum, situated approaches are important for the analysis of mobile navigation and use because they allow the identification of unplanned situations, commonly social in nature. Real-life situations can enable temporal tensions, interactions with objects and other people, problem identification, and avoidance in performing activities that are fundamental issues during everyday activities [22].

According to Ref. [29], research in Design seeks to understand the junction of different technologies and devices, the experiences of users in different life contexts, and the meanings that the use of a particular application or device provides for its users [27]. Expose the increase in the number of empirical and theoretical researches on the understanding of the phenomenon within a broader context and the realization of multi-methodological researches for mobile HCI investigations. As follows, research is being conducted within natural settings and on observation and using ethnographic methods, field experiments and field surveys [11]. In this way, has provided support for technologies such as embedded systems, smart spaces, and tangible interfaces, thus supporting evaluations of applications such as MobCare, as discussed in Refs. [40,41]. The reason for this is due to the need to expand interaction, traditionally performed with the fingers, eyes, or ears, to the whole body. Therefore, approaches need to provide support to assist and describe how these interactions occurred in detail [42].

In the literature it is possible to verify a growth in the number of studies with the same perspective, for example [43], with the performative prototyping method, which combines body storming methods with Wizard of Oz techniques. In Ref. [44] which introduces sociomaterial analysis of embodied design activities. [1], Presents the analysis of three research cases in which he analyzes the mediation of technique, embodiment and creative material engagement.

## 3 Materials and methods

The section will present the methodological details used to carry out the study.

### 3.1 Nature and approach

The definition of a scientific method comprises the declaration of the procedures that will be performed to obtain knowledge [45]. In this research, qualitative methods are adopted, aiming to understand the meanings of the context in which the object of study is inserted. The choice of the nature of the research occurred due to the alignment of the techniques used and the context analyzed.

The study was conducted using concepts from the phenomenological and postmodern paradigm. It was used concepts proposed by Digital Ethnography, in particular the re-enactment technique. Digital Ethnography comprises a contemporary approach to Ethnography, where the digital, material, and sensorial environments are considered. In this way, it explores and provides methodological support for understanding the presence of the digital in the everyday context, how they relate, the meanings that are attributed [46]. The “re-enactment” technique of Digital Ethnography, proposed by Ref. [47], comprises in the re-enactment of an everyday activity, in the situated environment with real elements that are part of the individual's life. Through the method it is possible to perform an immersion in reality and in the participants' knowledge. It allows participants to imagine and recreate the performance of a routine activity and reflect on their performance, identifying aspects of self-identity and embodied knowledge, obtained incrementally and shaped over time [47].

The technique was chosen due to the geographic and access limitations to the participants' homes. Most of the participants live in different municipalities and far from the capital. In addition, the use of some videoconferencing tool and local recordings during the real activity could obtain limited data due to the unfamiliarity of its use and due to the availability of digital resources to carry it out. The collection was carried out in the context of the Altino Ventura Foundation, during the break or the period of the child's treatment. It was possible to follow some of the mothers' routine with the children in the foundation, as well as to understand how the practices occur in the situated environment.

In addition to the ethnographic approach, qualitative research concepts were used, in which a Semi-Structured and Unstructured Interview script was created to guide the researcher during data collection (See Appendix 1). The choice of the technique occurred due to the possibility of establishing a basic script, however, as it is a situated approach new insights and new questioning were added during the interview enriching the data produced.

### 3.2 Context

During the analysis and creation of the study methodology, possible applications that would serve to analyze and evaluate the approach in question were analyzed. However, the MobCare application was chosen due to the knowledge and participation since its conception of some of the researchers of the study. The access to initial information and access to the field for data collection was possible and desired by both parties, since the results obtained by this paper can contribute to the evolution of the application and improvement in the quality of life of its users.

The context in which the application was developed was marked by the increase in the number of children with Microcephaly born to mothers infected with the Zika virus in regions of South and Central America and the Caribbean in the year 2015 [48].

The MobCare project began in 2016 with the goal of creating a tool that would help in the daily lives of caregivers of children with Microcephaly. To understand the context and the main needs, an analysis and immersion was conducted with the participants and professionals of the institution. The process consisted of interviews. As a result, the main difficulties faced were identified that served to develop a technological solution, which would establish a communication channel to assist in the monitoring and evolution of child care and contact with professionals from different specialties.

### 3.3 Prototype

**Fig. 1:**
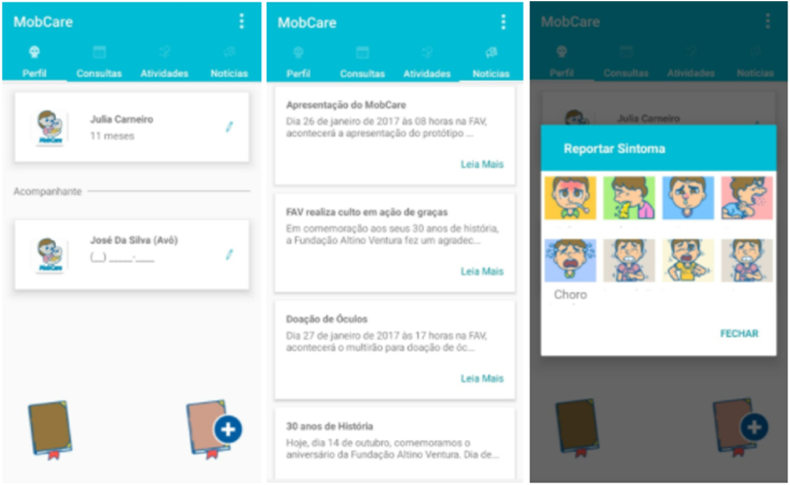
Mobcare application - altino ventura foundation.

MobCare is an intelligent platform for monitoring and integration of information related to the clinical pictures of children with Microcephaly (Fig. 1). The application aims to monitor the evolution of the children, monitor the symptoms of the diseases, and encourage and control the performance of therapy exercises.

● Allow access to short, illustrative videos that teach how to perform the therapies' stimuli at home;
● Report situations with the child;
● Report exercises performed;
● View upcoming appointments;
● View clinical situation;
● View and record information in the child's diary;
● Access information and news of the Foundation.

After the development was completed, no usability evaluation was done. Although it has been in use for over 2 years, it is necessary to understand if the initial assumptions and requirements identified continue to meet and help the daily lives of mothers.

### 3.4 Participants

The present study considered as a population the caregivers of children affected by Zika virus syndrome, i.e., mothers, fathers, grandfathers and/or people responsible for child care who are undergoing treatment at Altino Ventura Foundation. The selection of participants considers the following aspects: They were present at the Foundation on the days the data collection took place, they are users of the MobCare application, and they volunteered to participate in the study.

To declare the ethical aspects, the research project was submitted to the Ethics Committee and had its approval in December 2019. The Informed Consent Form was established to reinforce each participant's consent about the research.

The participants are characterized as mothers, have an age range of 28–39 years, almost all have complete high school education and an average of 2–3 children older than the child in treatment, as described in Table 1. Due to the necessary care with the child, they do not work and the family income is composed of a minimum wage obtained through some social program that the child is registered for or due to the father's work.

**Table 1:** Characteristics of the participants.

Participant	Age	N° of children	Work	Education level	Family income
Caregiver 1	35	2	No	Complete high school	Minimum wage
Caregiver 2	36	4	No	Incomplete Elementary School	Minimum wage
Caregiver 3	35	2	No	Complete high school	Two Minimum wage
Caregiver 4	39	2	No	Complete high school	Minimum wage
Caregiver 5	39	4	No	Complete Elementary School	Minimum wage
Caregiver 6	28	2	No	Complete high school	Minimum wage
Caregiver 7	34	3	Yes	Complete high school	Minimum wage

Due to the onset of the COVID-19 Pandemic in the year 2020, data collection cannot be continued and the present work could only rely on 7 collaborations. However, according to Ref. [[49], [50]][51], the quantification of a qualitative sample is subjective, usually related to the researcher reaching a point of redundancy and saturation of the observed information and behaviors. Due to the results obtained through the interviews collected it is possible to perceive saturation and similar findings, so we understand that the work can satisfactorily obtain the desired results and has relevance.

### 3.5 Design process

The immersion, design, and development of MobCare began in 2016, through observations and semi-structured interviews with mothers and professionals at the Altino Ventura Foundation. During the immersion activities, some points for improvement were identified, activities that could be optimized or that would help in the daily activities of the children's caregivers.

Initially, the scheduling of appointments was done by the professionals of the Foundation through the internal control system, however, the follow-up by the mothers was done through agenda cards. During the appointment days, they would go to the reception to update the cards with future appointments. Given this, as the agenda card was a physical object, a limited artifice, it could be lost or forgotten. Therefore, it was suggested the need to include a feature in the application that could replace the physical card and allow monitoring and alerts via cell phone.

Another point observed is the need for the child to receive stimuli outside the environment of the Foundation. During the interviews it was possible to identify the idealization of the creation of an instructional guide for the stimulation and care of children. The mother could consult and take away instructions and information presented during follow-up and treatment at the Foundation.

Through the results obtained, the first prototypes were developed, initially on paper and later with high fidelity.

**Fig. 2:**
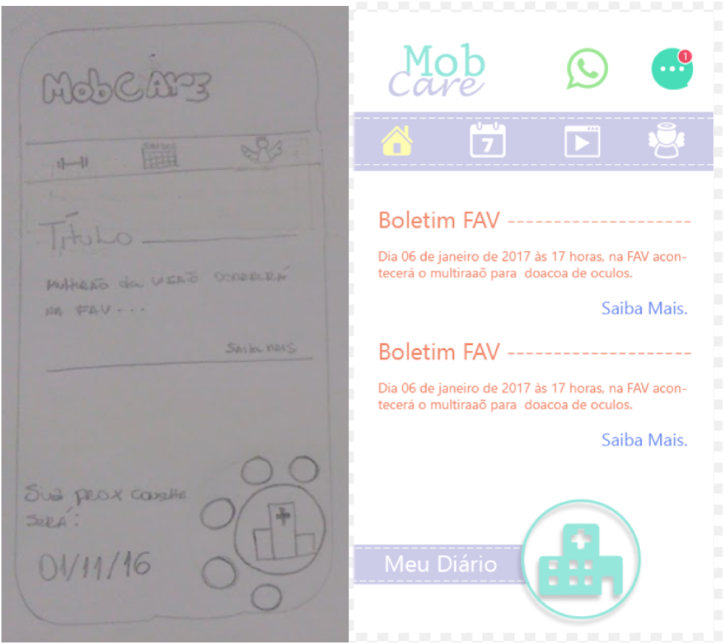
High-fidelity paper prototype of MobCare.

Over time, the application evolved (Fig. 2) with the inclusion of new features and changes in the interface, however, although during the study period the application had already been in use for about 2 years, it had never undergone a usability evaluation. In light of this, it is with this motivation that the study carried out the evaluation, aiming to explore and understand whether the application is still sufficient or which updates will be necessary.

### 3.6 Procedures and data collection

A functional version of the application was used to conduct situated prototyping. The methods employed paralleled techniques found in ethnographic and situated approaches. This version was utilized to instigate its use in a contextual setting, with our focus being directed towards understanding the implications of its use and the gaps that innovative improvisations might highlight. The research carried out data collection in March 2020 at the Altino Ventura Foundation, located in the city of Recife, Pernambuco, Brazil.

The procedures were executed individually, as the perceptions of one participant could potentially sway the responses of another. In the course of data collection, a Go-Pro camera, a smartphone equipped with the MobCare application, a screen recording application, and an audio recorder were employed.

The participations began by seeking to understand the life context in which the mothers and children are inserted. By using the situated approach, it is possible to follow the real, the routine, and the natural interactions and interventions that could only be observed in a similar context to capture the phenomena.

The initial interaction with the participant began with a welcome and thanks for her availability to contribute to the study. Then, the context, objectives and how the study would occur were presented so that if necessary there was the clarification of doubts or in case of discomfort the participant could withdraw from the study. The ethical issues were established and the interview for identification and characterization of the interviewee began.

Adopting a semi-structured interview script presented in the previous sections, the interview was initially conducted by characterizing the interviewee, identifying his/her relationship with digital and understanding a little about the stimulation routine according to the script sequence (Appendix 1). At the beginning of the “Using the MobCare application” section of the script, the use of the Digital Ethnography re-enactment technique was initiated. At that point, the participants were instructed to exemplify and recreate everyday situations with the MobCare application, being guided by the request of the researcher. During the realization, the researcher made a smartphone with the prototype of the application available to the participant to obtain the explanation aloud of situations with the application. At this moment, the researcher cannot help the participant to solve the questions about the application, because the questions, lack of knowledge, doubts, perceptions can be exposed during the explanation. The participant interacted with the following functionalities of the application: 1. Find notices from the Foundation; 2. Register symptoms; 3. Perform suggested stimulation using the videos; 4. View schedule; and 5. Access the child's diary. The observation of how the participants used the app during the re-enactments were noted through their speeches, the movement of their bodies, and their actions on the digital app.

The re-enactments of the activities consisted of the participants demonstrating how they perform the activity that the researcher asked them to perform in the same way they use it during their daily lives. It would then be possible to capture perceptions, meanings, and contingencies of use.

Finally, upon the need to further explore the data collected earlier, we conducted an unstructured interview during conversations with the participants to capture aspects of the experience of caring for their sons and daughters to understand if the app prototype matches their needs. The prototyping situation was perceived as another moment of knowledge construction.

### 3.7 Data analysis

The procedures described in the data collection section produced video, audio, and screen capture data. The data was analyzed incrementally and complementarity in building knowledge about the aspects that the situated approach provided. Because of this, re-analysis of the data and codes was performed as needed.

The first records analyzed were the audios, in which they register the dialogues about the reconstructed experience in the situated environment. The interviews were transcribed and with the text files, the thematic analysis was performed.

Next, the video data records the embodied behavior and actions of the participants during the interview and interaction with the prototype. After performing the transcriptions and audio coding of the interviews the analysis of the videos was performed. Through the corporeal and non-verbal aspects, it is possible to confirm the meaning of the verbal discussions and confirm the understanding about the participant's experience, as well as identify new themes that enrich and align with those identified.

Finally, the screen capture provides further support for the analysis of the effectiveness of the situated and embodied approach. Through it, it is possible to observe the efficiency of the use of the application, in a way that is aligned with the results obtained previously. Through the data obtained in the screenshots it was possible to evaluate the efficiency of the performance of a task. During the analysis the conformity of the instructions provided when demonstrating how to perform the activity are considered. The information will allow the identification of obstacles or problems in the use of the platform, causing the participants to fail to perform or have great difficulty in completing the tasks proposed in the third step of the procedure section. In keeping with the research goals, our aim was to discern facets connected to embodied experience, impromptu improvisations, contingencies, and meanings. We also sought to find additional evidence to strengthen the assertion that the situated and embodied approach is effective in the evaluation of a software prototype. The videos, the audios, and the screen captures served as input to evaluate whether the embodied and situated approach is effective in identifying the participants' perceptions, i.e., suggestions, improvements, criticisms, compliments, new visions, and ideas.

According to Ref. [48], one of the main challenges of the study of experience is to understand the media and what meaning, “affective and embodied”, it holds for an individual. It is occurring since the device, the interaction and the everyday situation cannot be separated. The phenomenological analysis is of utmost importance, as it allows us to understand which points of view, perceptions, meanings the individual attributes to a device [52].

The perception of context as being situated and embodied when audio and video recordings were made during the application of the interviews, it was possible to identify the main meanings that participants attribute to the practices of caring for their sons and daughters and the meanings attributed to digital artifacts such as the cell phone and the various applications used, including the prototype in the context of use.

### 3.8 Ethical aspects

To declare the ethical aspects, the research project was submitted to the Ethics Committee of Altino Ventura Foundation through Plataforma Brasil. It was approved in December 2019 with Certificate of Presentation for Ethics Assessment (CAAE) number 26566619.7.0000.5532.

## 4 Results

The significance of the current application and especially the strategies to navigate through the contingencies of conventional uses represent the most notable outcomes of our prototype's effectiveness. The interpretation of these data aids in steering the design process towards a solution that aligns the concept more closely with what is contextually relevant. We analyzed the essential activities in the daily life of mothers. Their days start quite early, often beginning by organizing tasks for their husbands and children. Many reside in towns far from the Recife Metropolitan Region, such as Aliança (82 km) and Condado (58 km). The displacement occurs practically every day as the child undergoes treatment at the Altino Ventura Foundation and other institutions in the capital. Typically, they usually use the transport provided by the city hall or government program or go to the bus stop to take public transportation.

On the days that there are no appointments, the mothers divide their time with the daily care of the home and care of the child, performing stimulation or taking the child to school. It is usually during this time when they are at school that they are able to perform other personal and professional activities.

Digital resources are always present in everyday environments. Through the reports it was possible to identify the presence of devices such as televisions and especially cell phones and smartphones in the participants' daily lives. In relation to the utilization of technological tools and devices, the use of mobile phones emerged as the primary companion for various activities. This includes scheduling appointments, access to information and social networks, contact with family and leisure, a source of stimuli and fun for the child. The device usually accompanies mothers throughout the day. However, its use is generally conditioned by two main factors: time availability and internet access. The daily life of mothers is very hectic and sometimes they cannot allocate so much time for the use of the cell phone, however whenever they can they are looking for updates through it. The second factor is related to internet availability. When they are at home, the main source of internet is broadband with Wi-Fi access, while to access information during the day outside the home 3G is used.

“So I don't have much time not to be in these things, but I use it more for information. In this case, an appointment was rescheduled or will not have an appointment. It's for the communication use of a group we are part of. Information, more for that. To talk to the family."- [Interviewee 5].

In addition to access to information related to the children's conditions, it is also possible to observe that they maintain a social interaction, between mothers or with the hospital staff, through social networks and messaging applications such as Facebook, Instagram and WhatsApp. Digital social networks are appropriated and their meanings for the participants in childcare practices within many others, as revealed:

“With the cell phone I do research. I work with sales too. I access Facebook, Instagram, WhatsApp" - [Interviewee 7].

It is also interesting to note the strong relationship they have and the communication extends to the halls of the Foundation and even to the digital media. Besides the integration groups that the Foundation creates, it was reported that they have groups to exchange experiences and support. It was possible to see mothers together talking in the hall, or even how they could contribute to this study, indicating who was in the same group that could help. These interactions continue on the digital communication platforms and do not have well-defined boundaries. Throughout the interviews, we noticed a preference for social networks, including Facebook, Instagram, and WhatsApp. WhatsApp was the most frequently mentioned app, serving as the main channel for acquiring both personal and professional information, as revealed: *"Direct, I think about 12 hours a day; I'm on the internet. Why not just on the internet, why the "Zap", my "Zap" is like life". […] "For me, it is essential. I was once asked like this … Can you go for a week without your cell phone? Oh, I said I can, but the cell phone can't. It's not me who can't; I could if I didn't have it (child's name) because the whole world, my daughter's world, is turned to a cell phone." - [Interviewee 3].*

Given that the app wasn't explicitly designed for medical communication purposes, we could surmise that the observed adaptation signifies an improvisation in line with [46]. Users create senses and meanings that emerge from experiences in contexts of use.

In summary, digital platforms form an integral part of activities that consistently come to life between individuals in their day-to-day lives. They are pervasive in numerous practices associated with child care and crucial for keeping track of activities of those around them. The device appears to symbolize a guarantee of access to communication and vital support from the health department, as well as other mothers who are the bearers of opportunities and hope for treatment and advice. As can be seen in the above account, Interviewee 3 comments on WhatsApp being present 12 h a day in her routine. It was observed in other mothers' reports. Currently, WhatsApp is one of the most used communication tools among them. Through it, it is possible to participate in many activities, for example, such as scheduling appointments and communicating with professionals about events with the child.

“Then we always have to see because the doctors always send consultations, exams, the scheduling of an exam. We always have to be connected, because nowadays everything is on WhatsApp, right? - [Interviewee 6].

Communication expands the possibilities of monitoring by the community of health professionals and family. However, we note that this communication is unstructured. Reconstructing dialogues and actions linked to posts in the form of the WhatsApp textual genre evolves but is nontrivial for family members. Therefore, we perceive this appropriation as a creative improvisation that points to several functionalities that can be considered essential to improving the experience of individual monitoring of children. Examining the usage of the current MobCare prototype by mothers, it appears that its application is typically conditioned by the necessity to document the incidence of a symptom or illness. This seems to induce users to only register negative symptoms, which can bring a negative memory with the application, and all communication functionalities that could be done through the tool ended up being registered outside the application:

“I use it once a week, twice. It depends, why I use it more, when she doesn't feel well." […] "In my daily life yes. When I see the need to … to pass on what is happening in the day to day with her. If she is unwell, if she had a fever, if she didn't have a fever, if there is something, I have to put it in the application to receive orientation since I am far away, right? - [Interviewee 6].

“I don't use it much, because she's not a seizure child. It's very hard to get sick too." […] - [Interviewee 3].

Through the contextual analysis it was possible to observe that some functionalities of the application were not incorporated in activities among users, or even perceived by them. We noticed when during a re-enactment we asked to simulate the use. We expected to observe the use of the news functionality or the journal view. However, this manipulation was not observed in the re-enactment. It may reveal a lack of appropriation of part of the functionalities prototyped for the application, despite the fact that the mothers have been using the application for about two years, at the time of a public presentation of the functionality:

“How do you normally do to watch the news?" - [Researcher].

“If he has a fever, then I come here and press "fever". Then I put the date, day and time. If he is vomiting, I do the same thing, I mark everything here, the number of times he vomited per day. [ …]" - [Interviewee 5].

The interpretation can be twofold: problems with usability and/or a lack of complementary requirements/functionalities that allow complete communication through the tool.

Another functionality designed in this prototype to supplant essential needs of activity taken as essential for families was to report the child's symptoms. The design had the intention of allowing the collection of information at the moments they occur and thus serve as a platform for collecting and identifying patterns of chart evolution. For which, in view of what was observed, it was possible to conclude that the contingency may be related to technical limitations in the application, for example, the description field in the symptom record has a limitation in the amount of characters registered. Although the mother can type a large text, the professionals can only visualize small excerpts, as highlighted in the speech of interviewees 3 and 4 below. It was possible to observe their yearning for improvements to this problem, because although they have found a creative solution for sending messages, this problem interferes with the time to post an event.

“There is this difficulty. When we press the choke button, we have to say the time, then when we send a text, it has to be a little text … a little text. Then I send a little piece, then I send a little piece, then I send a little piece. - [Interviewee 3].

“It is in this part that the text is short […] Here, we write here, then write, she said that we have to write some little sentences. […] She wants me to write like this, (name of the child), with a fever of 38 and vomited. There. Very tiny. Because she can't see the bottom part" - [Interviewee 4].

As a result, it was observed that the participants devised a creative solution to circumvent the character limit and communicate with professionals. Those responsible for the children can share small excerpts and repeated records with professionals. Although it is an effective creative solution, it is possible to see the impact on use and adherence to use:

“It's difficult because we have to open the application every time and send it, then it goes back to the home screen, then it sends and goes back to the home screen. And that's it. We don't have much time and little time you already have to write something that is happening … " - [Interviewee 3]

Another impact of the practical usability relates to the consultation feature, as mothers report that not all types of consultations carried out at the Foundation are logged in the application. Consequently, we were able to observe additional creative solutions for follow-ups, such as monitoring upon arrival at the reception or through the use of diaries.

“The consultations don't show up. It shows up, so if it's with the physical therapist, the therapies, but consultations that I have with trauma, with the neuro, it doesn't show up [ …]." - [Interviewee 4]

It is possible to observe another improvisation in the follow-up of the queries by the application:

“The appointments I only follow up when we arrive at the reception desk. I've never followed up much on the application, the staff when I get here, they always call us, they call to let me know and we confirm at the reception." - [Interviewee 1]

“[…] I already take the agenda. There it is to see the schedule of things, the dates [ …]" - [Interviewee 2]

Another perceived functionality comprises notifications. Many applications commonly generate alerts for a variety of purposes, like reminders, newsletters, and promotions. They usually help and stimulate the use of an application. According to the mothers' feedback, MobCare does not have notifications, and due to the absence of this requirement, engagement is low due to their care routine. It is clear that the act of notifying could help mothers' engagement, as this would make them perceive it as an active communication tool.

“Yes, it doesn't arrive, we have to open the application to know when something arrives because it doesn't arrive, on WhatsApp it arrives when it vibrates, it rings, something, but it doesn't arrive. So, I see the difficulty that many mothers have in not being able to open it because we don't have enough time to do so. And it ends up going unnoticed, an event goes unnoticed [ …]" - [interviewee 3]

In summary, in the face of all the observation and results obtained from the contextual and embodied analysis of the use of an application, one can understand about the impressions and perceptions that emerge during its use. We conclude how the participants perceive the impact of the use of the application on their daily lives:

“I think it has improved. The only thing I miss is that they said they were going to put everything there. It doesn't have the consultation parts, right? It's not written down." - [Interviewee 4].

“So … I think it is good. Né? There is the part where you send a text message. There is the part where you mark, if he had a fever, then I go there and write it down [ …]" [Interviewee 5]. - [interviewee 5].

“I think it was a help, of support, sometimes … many times we want to clarify a doubt here and we are directly with the doctor and speak directly with the visual stimulator, psychopedagogue and she helps us. - [Interviewee 1].

No, generally MobCare helped a lot, because through this … Because for some it may even be simple, right? But only through this, I can already qualify if (name of child) spent the day well or not […] - [Interviewee 2].

Yes, there was improvement, because at all times that I contacted the application I receive immediate guidance on how to proceed. - [Interviewee 6].

## 5 Discussion

Through the situated and embodied approach, it was possible to expand the limits of the data traditionally obtained by audios and understand the dynamics and body experience in the face of the proposed situations. It is evident that the characteristics and abilities of the body and the environment contribute to the construction of understanding, rescue of the phenomenon and interactions performed. The use of the approach allows us to analyze the participants' interaction with the application, but also to obtain data related to the social environment in a situated environment.

The observations made during the collection period, together with the reports obtained through the interviews, allowed us to understand a little about the participants' routines, how they occur, factors that help in some essential activities, which objects are present and how they interact with them, what meanings they attribute. It was possible due to the theoretical and methodological choices, as it was combined with anthropological methods, such as Digital Ethnography, discussed by Ref. [46], and the observations made at all times at the Foundation. Another fundamental factor for understanding the aspects that make up the daily life and context of the participants is the observation of the interactional environment built by the relationships and their subjective characteristics. In this way, the bond and familiarity with the environment provide a feeling of belonging and collaboration with the necessary conditions for the realization of the understanding of the environment situated between embodied beings and the objects present in it.

In short, in the meaning of [28], the mothers' routines were very busy and the main environment in which routines occurred comprised the Foundation's premises. However, it was possible to understand the dynamics of the home environment before and after the treatment day. The analysis of the results obtained allows us to conclude that the use of the situated approach is effective in obtaining unpublished data for understanding about the cognitive experience of the participants in relation to the use of the application.

Through the literature review it was possible to observe that although there are many techniques and methods that help to perform evaluations of mobile applications, it is still discussed “where” and “how” to perform them [27]. As discussed by Ref. [22], this occurs due to the difficulty of capturing the dynamism and experiences of interactions with contextual factors that need to consider social, psychological, physical aspects, among others.

The results of the study reinforce the perspective of [22,28,51]. They defend that field studies, situated and embodied, allow the generation of more precise conclusions than those observed by other studies. Approaches such as ergonomics and having a greater ecological validity. Furthermore, it aligns with the thoughts discussed by the third wave of the IHC, which highlights the need for more humanistic analyses related to everyday aspects, as mentioned by Refs. [15,52]. The embodied aspects further reinforce the understanding of practical experiences and events. It confirms the theoretical premises of Embodied Cognition, which emphasizes the importance of physical interaction for the construction of cognitive processes, as discussed by Refs. [29,30]. Through the data analysis it was possible to understand the meanings attributed and obtained through the participants' body memory.

Furthermore, the approach applied by this study made it possible to overcome some challenges that mobile software evaluation has. Through the establishment of the procedures performed during data collection and the quality of the data obtained at the end of the analysis process, we can indicate that the methodology is effective in evaluating the application by applying the approach in the studied environment and contributes to the evolution of establishing a methodology suitable for the context of mobile applications. Furthermore, it can be observed that the use of the situated approach allows us to overcome the challenge of context analysis and ecological validity, which is fundamental to drive the design and evaluation of an interaction, as discussed by [53].

## 6 Conclusions

The speculation about the use of situated evaluation in the usability evaluation of mobile applications was presented. It aims to understand the applicability of adopting such theoretical and methodological postures.

Based on this work, we highlight four practical challenges that the situated and embodied evaluation technique can help solve: Methodology of situated and embodied evaluation: the development of the methodological roadmap contributes to the establishment and recommendations for applications of the study in other situated environments with applications from different contexts; Context and ecological validity: how the application of the situated methodology in one environment provided a high degree of ecological validity; Usability: through the evaluation of the embodied and situated it was possible to identify reports on usability problems; Understanding in the holistic nature: approaches encounter difficulties in analyzing the user and application simultaneously. Through the proposed approach it was possible to identify aspects present in the entire environment surrounding the evaluation, allowing more real and complete data to be obtained.

At the end of the study, it is possible to identify relevant aspects that can serve as a future path for the continuation of the discussed theme. Mainly because the current approach is aligned with the thoughts and characteristics of the third wave of the HCI, which is constantly evolving with the emergence of new methods.

Upon concluding the study, we are able to identify key aspects that may serve as a future direction for the continuation of the discussed theme. This is especially because the current approach is aligned with the thoughts and characteristics of the third wave of the HCI, which is constantly evolving with the emergence of new methodologies. The initial point involves the application of the study in another context and environment in a way that allows capturing new situations and experiences differently. In addition, performing the analysis over a more extended period in a way that allows the researcher a greater immersion in the culture of the group under observation. Complementarily, carry out a comparative study using a traditional and proposed approach. In addition to performing transcriptions that consider more linguistic and human data for analysis.

## 7 Limitations

Given the conduction and the results obtained at the end of the study development, it is possible to identify the main difficulties and limitations. The first point is related to data collection. Due to the routine of the mothers it was difficult to get time to present the research, talk and observe the routine of consultations, because everything happens in short intervals of time. In addition, some were more resistant to the interview and the need to use the camera. However, with the support of the Foundation's professionals, it was possible to carry out the study and obtain the expected results.

Another important aspect is related to the limited literature. Although the literature on situated cognition, situated interaction is already quite consolidated, its use for mobile application evaluations is still quite limited. In this way, there were difficulties to analyze similar approaches.

## Data availability statement

The data collected is confidential. However, anonymized data may be available upon reasonable individual request with the approval of all authors.

## Author contribution statement

Júlia Nogueira; Alex Gomes; Amadeu Filho; Fernando Moreira: Conceived and designed the experiments; Performed the experiments; Analyzed and interpreted the data; Contributed reagents, materials, analysis tools or data; Wrote the paper.

## Additional information

No additional information is available for this paper.

## Declaration of competing interest

The authors declare that they have no known competing financial interests or personal relationships that could have appeared to influence the work reported in this paper.

## Appendix A

### Interview Script – MobCare

#### Presentation

- Introduction of the researcher and greetings.
- Thanking the participant;
- Requesting permission to record (in case the participant does not consent to recording, all the following steps should be recorded in writing).

#### Introduction

The objective of this research is to understand the effectiveness of using a situated evaluation technique of software prototypes in face of the application Mobcare, a platform for real-time digital collection and monitoring of clinical and functional information and ophthalmologic diagnoses in the follow-up of the visual rehabilitation process of children with microcephaly, the Mobcare.

All information provided in this interview will be treated confidentially. Only the research team will have access to the information provided.

Your participation in this research is voluntary and you can decide not to participate or to withdraw from the research at any time. If you decide not to participate, you will not receive any sanctions or penalties.

- Do you agree to participate in this research?

#### Interviewee Identification

The following information will be used in case the research team needs to contact you in the future for clarification about the interview.

- What ‘s your name?

#### Characterization of the interviewee and child

- How old are you?
- How old is the child?
- Do you have more than one child?
- Do you work?
- What is your family income?
- What is your level of education?
- In which city and neighborhood do you live?
- How do you get to the Foundation on the day of your appointments?
- Is the child seen in more than one institution?

#### Relationship with the digital

- Do you use a cell phone?
- What model do you use?
- Do you access the Internet?
- Where do you get access to the Internet?
- O que você utiliza no celular?
- Do you use any applications? Can you name some examples?
- In a complementary way, do you think that using a cell phone is something essential for your daily activities?

#### Routine execution of the stimulations and use of the Mobcare application

In the next few questions, we will talk about your daily routine. Please try to relate your answers to practical aspects of your daily life associated with your child's care. As well as the use of the monitoring application, MobCare.

- Please, could you comment on your routine?
- Are you or someone else responsible for the child in the habit of providing stimulation to the patient outside of the hospital environment?
- How often do you do the stimulations?
- Are there any factors that hinder the performance of stimulation? If yes, which ones?

#### Using the Mobcare application

- In your daily life do you use the MobCare application? If so, could you comment a bit on it?
- If yes:
- What improvements can you highlight from the period when you did not use the app and after using it?
- How often do you use the application?

#### [Find Announcements]

- How are you informed about the notices?
- If you use MobCare:
- Please, could you show me how you see the notifications?

#### [Register Symptoms]

- During the routine, many times the child may present some symptoms, correct? How do you notify the Foundation staff of what has happened?
- If you use MobCare:
- Please could you show me how you do to register a new symptom?

#### [Perform suggested stimulation using the videos]

- As mentioned earlier, it is necessary to always carry out stimuli with the child, is it difficult to remember the instructions provided outside the foundation? How do you carry them out?
- If you use MobCare:
- Please could you show me how you do it to get more information about the stimuli?

#### [View Schedule]

- How do you keep track of scheduled appointments?

#### - If you use MobCare

- Please could you show me how you do to keep up to date and keep track of scheduled days and times?

#### [Access Child Diary]

- Do you have any control or access to your child's diary?
- If you use MobCare:
- Please could you show me how you do to access your child's journal record over the course of the reminder?
